# Electrophysiological evidence of the amodal representation of symmetry in extrastriate areas

**DOI:** 10.1038/s41598-021-04501-3

**Published:** 2022-01-21

**Authors:** Giulia Rampone, Martyna Adam, Alexis D. J. Makin, John Tyson-Carr, Marco Bertamini

**Affiliations:** 1 Department of Psychology, University of Liverpool, Eleanor Rathbone Building, Liverpool, L697ZA UK; 2 Department of General Psychology, University of Padova, Via Venezia, 8, 35131 Padova, Italy; 3 School of Psychology, University of Liverpool, Eleanor Rathbone Building, Liverpool, L7 7DL UK

**Keywords:** Extrastriate cortex, Visual system, Object vision, Perception

## Abstract

Extrastriate visual areas are strongly activated by image symmetry. Less is known about symmetry representation at object-level rather than image-level. Here we investigated electrophysiological responses to symmetry, generated by amodal completion of partially-occluded polygon shapes. We used a similar paradigm in four experiments (N = 112). A fully-visible abstract shape (either symmetric or asymmetric) was presented for 250 ms (t0). A large rectangle covered it entirely for 250 ms (t1) and then moved to one side to reveal one half of the shape hidden behind (t2, 1000 ms). Note that at t2 no symmetry could be extracted from retinal image information. In half of the trials the shape was the same as previously presented, in the other trials it was replaced by a novel shape. Participants matched shapes similarity (Exp. 1 and Exp. 2), or their colour (Exp. 3) or the orientation of a triangle superimposed to the shapes (Exp. 4). The fully-visible shapes (t0–t1) elicited automatic symmetry-specific ERP responses in all experiments. Importantly, there was an *exposure*-dependent symmetry-response to the occluded shapes that were recognised as previously seen (t2). Exp. 2 and Exp.4 confirmed this second ERP (t2) did not reflect a reinforcement of a residual carry-over response from t0. We conclude that the extrastriate symmetry-network can achieve amodal representation of symmetry from occluded objects that have been previously experienced as wholes.

## Introduction

The visual system is highly tuned to symmetry, both in humans^1–7^ and other animals^8–10^. By definition, symmetry is a non-accidental property characterized by rigid transformations^6,11,12^. Among the different types of transformations (i.e., reflection, rotation, translation), reflection is the most salient to the visual system^13,14^ and is considered a fundamental cue for figure-ground segmentation^15–19^. According to formal models of symmetry, perception of reflection symmetry relies on the analysis of pairwise correlations between elements along a central axis^6,11,12,20–25^.

**Figure 1:**
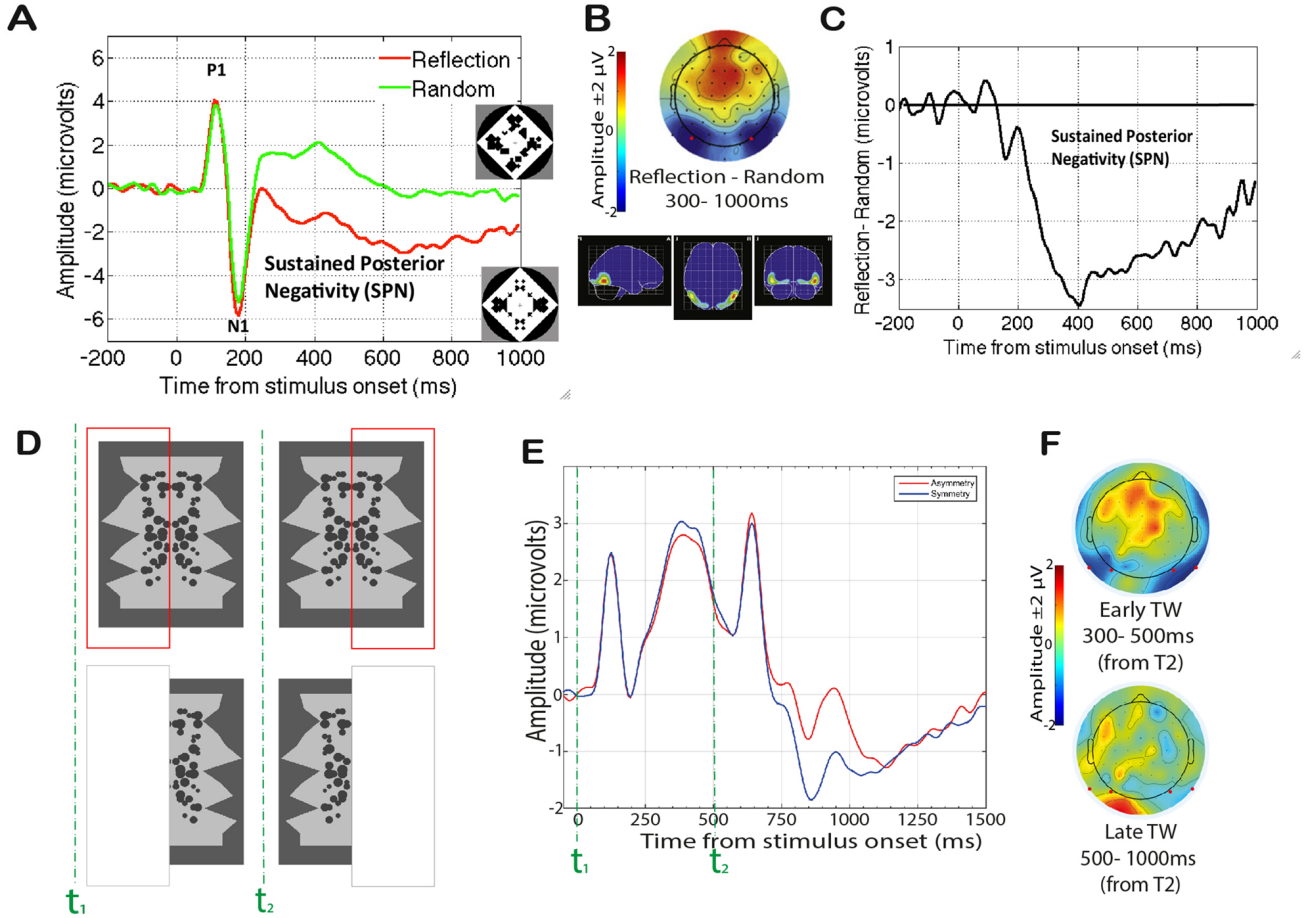
Sustained Posterior Negativities from previous research. (**A**–**C**) Results from Makin et al.^42^, Experiment 1 (**A**) Grand average ERP averaged across electrodes PO7 PO8 in reflection (symmetry) and random (asymmetry) conditions. Example stimuli are shown in insets. (**B**) Topographic difference map from 300 to 1000 ms post stimulus onset (SPN is coded as blue at posterior electrodes) and estimated cortical sources of the grand average SPN. (**C**) Grand average SPN shown as a difference wave (symmetry–random). (D-F) Stimuli and Results from Rampone et al.^51^, Experiment 1 (**D**) Examples of stimuli (symmetric shapes). The example on the top reports the full shape; the red bar mimics the actual occluder bar presented in the experiment. On the bottom, example of the same shape with the occluder. (**E**) Grand average ERPs averaged across electrodes P9 PO7 P10 PO8. From 0 ms (t1) to 500 ms the first half of a polygon shape was visible. At 500 ms (t2) the occluder moved and revealed the hidden half, whilst covering the previously visible part. After ~ 300 ms, a symmetry–asymmetry ERP was observed. (**F**) Topographic difference maps (Symmetry–Asymmetry) from the time-window 300–500 ms and 500–1000 ms from t2. Red dots indicate electrodes analysed.

The neural basis of symmetry detection has become clearer in recent years^26,27^. Symmetry is processed in extrastriate regions—V3, V4 and Lateral Occipital Cortex (LOC)^28–34^, whilst areas V1 and V2 do not show any sensitivity to symmetry^32–34^. Importantly, LOC plays a causal role in symmetry detection^35,36^. Electrophysiological (EEG) measures have established an event-related potential (ERP) index of symmetry representation. This is called the Sustained Posterior Negativity (SPN)^37–42^ (see Fig. 1A–C). SPN is recorded over posterior electrodes and is likely to be generated by the extrastriate symmetry network^39,42^. The SPN begins around 250–300 ms after stimulus onset and is sustained even beyond stimulus offset^43^, if there is no mask. SPN amplitude scales with the perceptual goodness of the stimulus: the more salient is the regularity the larger the amplitude^39,44,45^. The SPN is task-independent: it is generated when symmetry is present in the image, even when attention is directed to other stimulus features (e.g. colour^30,41,46^) or different stimuli in different modalities^43,44,47,48^ . The SPN is a difference wave: it isolates the differential brain responses to symmetrical and asymmetrical stimuli (Fig. 1). However, the SPN wave is generated whenever local element position information is combined into a global gestalt. SPN-generating gestalt formation processes also happen when viewing Glass patterns^49^ or when viewing line drawings of familiar objects^50^.

Recent research has investigated the neural representation of symmetry at *object-level*, i.e. when symmetry is not in the image, it must be extracted by means of specific computational processes^30,46,51,52^. For example, the symmetry-network can adjust for changes in perspective (i.e. 50° slant), leading to a view-invariant SPN response^46^ (see also similar fMRI evidence^30^). Symmetrical stimuli can also be partly occluded, or different parts of the symmetry can be seen at different points in time. In such conditions, symmetry is never present in one retinal image. Despite this, an extraretinal, object-level, representation of symmetry can be selectively constructed when it is relevant for current tasks. These object-level symmetry representations generate an SPN. For instance, Rampone et al.^51,52^ found symmetry-SPN response formed through integration of parts presented at different intervals with dynamic occlusion, although this was not sustained (**D**–**F**). These studies demonstrated the flexibility of the symmetry representation.

It is interesting that no symmetry-asymmetry response is observed from static occluded abstract polygons (see Fig. 1E, t1–t2). On one hand, this is not surprising because image information is similar (and asymmetric) in all conditions. On the other hand, the experience of occlusion may induce a tendency to *amodally* complete the hidden part of the shape. Amodal completion is the process of completing objects in the absence of direct visual sensory input in the occluded region^53–55^. The occluded information is *filled-in* leading to a holistic representation of the complete object. In the brain, amodal completion is achieved in extrastriate regions (i.e. LOC and Inferior Temporal (IT) cortex)^56,57^, which are implicated in object recognition.

Generally, amodal completion is guided by either local cues (e.g. good continuation or T-junctions), leading to the simplest possible completion process^58,59^, or global cues (e.g. shape regularity, symmetry), leading to the simplest possible completed shape^60–63^. These cues can lead to different competing interpretations^62,64^. Several studies have shown that global completion (i.e. tendency to maximize the symmetry of the occluded object) tends to be the default, or preferred, mode of interpretation^65–70^. However, the selection of the preferred interpretation may be influenced by different factors^62^. For example, when occlusion covers a substantial portion of the shape (i.e. half), the vertical axis of symmetry is not a dominant factor for global completion, and local completion may be preferred^62,71^. Note that for the polygons in Fig. 1C, global completion would depend on vertical axis of bilateral symmetry.

Several studies have shown that top-down influences may mediate the amodal completion process. Global completions are less precise than local completions and, therefore, may rely on object knowledge^72–77^ (but see^78^), object familiarity^58,79,80^ (but see^59^), surrounding objects^81^, and preceding objects^76,82^ based recognition of partial information of the occluded figures^77^.

Recent *prior exposure* to a novel shape can influence the amodal completion of the same shape presented again behind an occluder^75,76,79^. Hazenberg et al.^79^ found that this is especially true for cases of greater uncertainty (i.e., when the visual properties of the occluded shape favour multiple completions; see also^78^). Prior exposure can induce completion interpretations that are otherwise unlikely^76,83,84^. Plomp and van Leeuwen^82^ used a paradigm in which exposure to single complete figures biased the completion of composite figures presented after a short interval, but only if they were congruent (i.e. if the first was a possible interpretation of the second).

### The current study

In four experiments we investigated brain responses to the symmetry of partially-occluded objects (i.e. object-level representation), through amodal completion processes.

**Figure 2:**
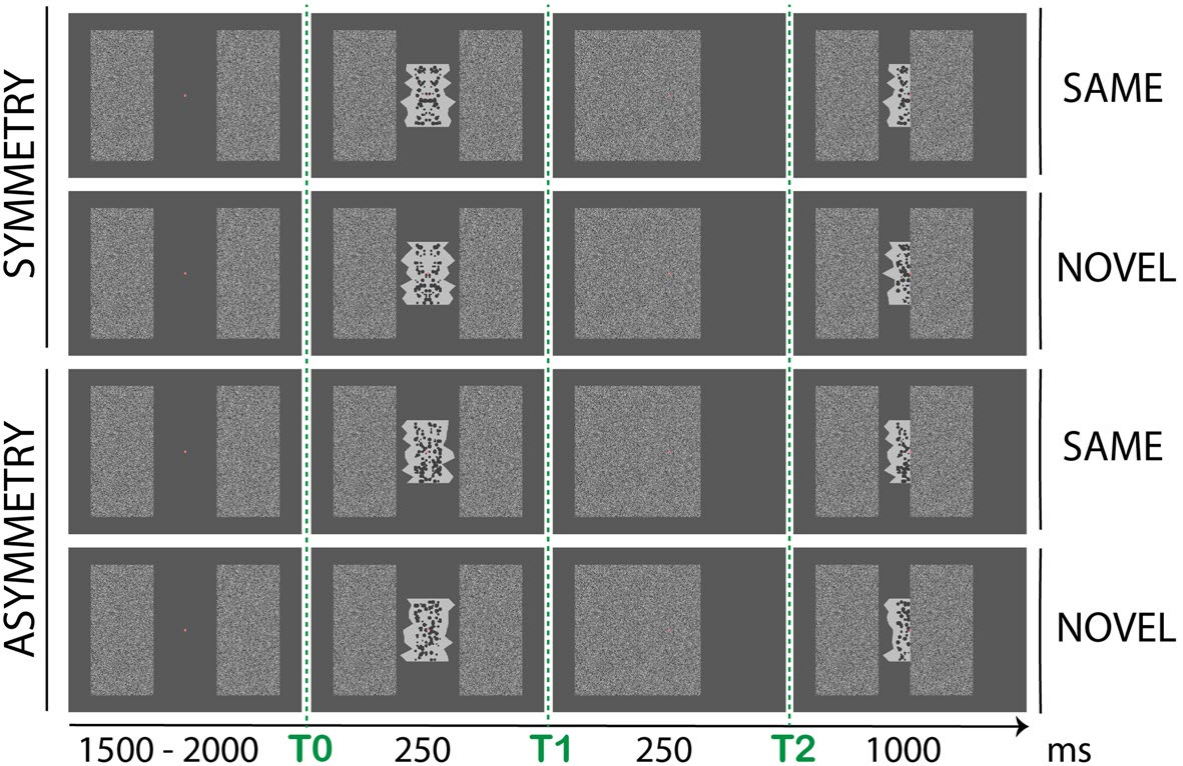
Experiment 1. Example of stimuli and experimental procedure. A fully-visible abstract shape, either symmetric or asymmetric, was shown for 250 ms flanked by two large rectangles (t0). One of the rectangles (the right on in the figure) moved towards the center to cover the shape. The occluder then moved back (towards the right) to reveal half of the shape underneath (t2). The now partly-visible shape may be the *Same* as previously seen or a *Novel* (different) shape. The four possible conditions (from top to bottom) are: *SymmetrySame*, shape in t0 is symmetric and shape in t2 is the same (but half-occluded); *SymmetryNovel*, shape in t0 is symmetric and shape in t2 is different (novel); *AsymmetrySame*, shape in t0 is asymmetric and shape in t2 is the same; *AsymmetryNovel*, shape in t0 is asymmetric and shape in t2 is different. For simplicity, the figure illustrates only displacement of the right-occluder. The same combinations were obtained for the left-occluder (counterbalanced across trials).

We designed a paradigm where participants first saw a full abstract shape (t0, 250 ms), either symmetric (one-fold bilateral reflection) or asymmetric, flanked by two large rectangles. The following timeframe gave the impression that one of the rectangles moved towards the center to cover the shape entirely (t1, 250 ms). The occluder then moved towards the original position to reveal only half of the shape underneath (t2, 1000 ms). The partly-visible shape could remain either the *Same* as previously seen or be a *Novel* (different) shape. Figure 2 illustrates the four possible conditions (from top to bottom): *SymmetrySame,* the shape in t0 was symmetric and the same shape was presented half-occluded in t2; *SymmetryNovel,* the shape in t0 was symmetric and a novel shape was presented in t2; *AsymmetrySame,* the shape in t0 was asymmetric and the same shape was presented in t2; *AsymmetryNovel,* the shape in t0 was asymmetric and a novel shape was presented in t2.

Note that in t2 all conditions were similar and asymmetric. The hypothesis was that prior exposure to a symmetric whole would influence the global amodal completion of its part presented in a second timeframe^82,85^.

To avoid confusion, we should mention a phenomenon called *SPN priming* (i.e. increase in SPN amplitude with presentation of symmetrical images in succession^86–88^). This form of past-history effect reflects interdependency of responses to fully-visible image symmetries. This is quite different from the current study. An independent symmetry-response was expected in t2 for the *SymmetrySame* condition, reflecting object-level representation of the partly-occluded symmetric object.

## Experiment 1

Twenty-eight participants took part in this experiment. The task was to report whether the half-occluded shape in (t2) was either the *Same* as the previously seen shape or a *Novel* shape (see Fig. 2). Note that the symmetry of the shapes was never made explicit to participants before final debrief. This experiment tested the presence of a symmetry-specific posterior negativity generated by the completion of the half-occluded shape. We expect that this form of amodal completion would only emerge after prior exposure to the whole shape^62,75,76,79,82^.

Because the SPN is a relative measure of the difference between ERP response to symmetry–asymmetry, we analysed the following SPNs: *SymmetrySame–AsymmetrySame* and *SymmetryNovel–AsymmetryNovel*. Our predictions involved two distinct timewindows. We expected typical SPN after presentation of the whole shapes in t0 (i.e., both *SymmetrySame–AsymmetrySame* and *SymmetryNovel–AsymmetryNovel*), because we know the brain automatically responds to symmetry in the image^43,46–48^. This response was expected to be short-lasting due to the onset of new stimuli. In t2 (when shapes were half-occluded) we expected a second posterior negativity to emerge, reflecting the amodal completion of the composite shapes. This completion should only emerge for shapes that were same as those previously seen (i.e., *SymmetrySame–AsymmetrySame*).

We expected no *SymmetryNovel*—*AsymmetryNovel* difference because prior exposure to the global interpretation was deemed as necessary (otherwise local completion should be preferred^62,71^). Timewindow analysed was 800 ms–1000 ms (i.e. 300–500 ms from t2); this was chosen a priori based on Rampone et al.^51,52^ and it best indexes the neural correlate of symmetry perception^39,51,89^. The electrodes-cluster analysed (P9, PO7, P10, PO8) was also selected a priori^51,52^.

### Results

**Figure 3:**
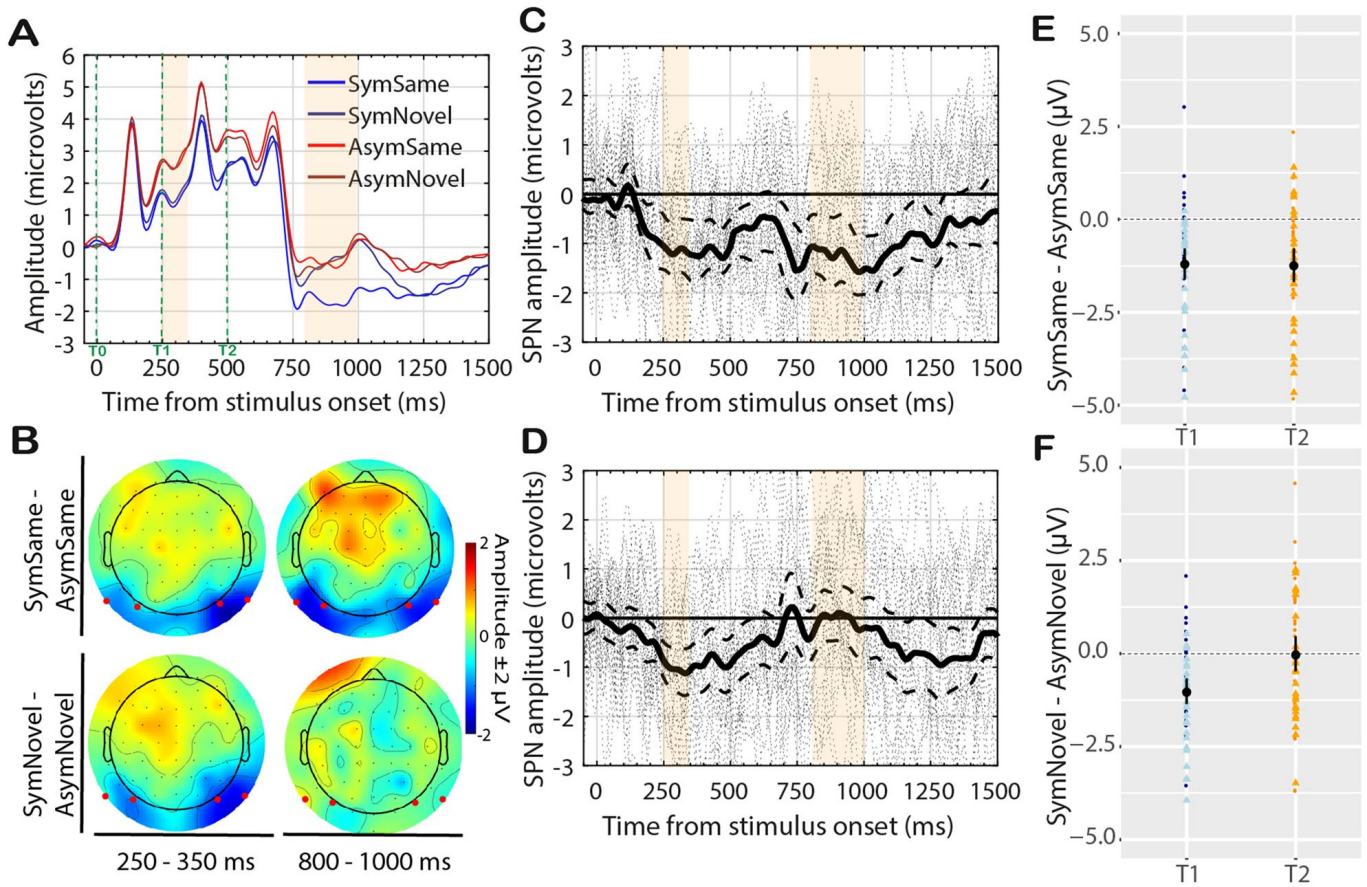
Experiment 1 results. (**A**) Grand average ERPs averaged from electrode cluster P9 PO7 P10 PO8. A SPN is observed after ~ 200 ms from T0. Then SPN emerges again ~ 250 ms from T2, only for the SymmetrySame condition. The orange regions indicate the time-windows used for the analysis. (**B**) Topographic difference maps (Symmetry–Asymmetry) for the time-window 350–350 ms and 800–1000 ms from T2. Red dots indicate electrodes analysed. (**C**) The SPN (difference wave) for *SymmetrySame*–*AsymmetrySame* and (**D**) the SPN for *SymmetryNovel*–*AsymmetryNovel*. Solid line is shown along with 95% confidence intervals (C.I.; thick dashed lines) and individual-subject responses (thin dashed lines). When C.I. are below zero, the difference wave is significant at the 0.05 level. (**E**) Stripchart (i.e., one- dimensional scatter-dot plots) showing distributions of individual difference amplitudes for *SymmetrySame*–*AsymmetrySame* and (**F**) *SymmetryNovel*–*AsymmetryNovel* at the two timewindows. Dark-coloured dots represent responses at the left hemisphere; light-coloured triangles represent responses at right hemisphere. Mean difference amplitude is superimposed (black dot), and error bars indicate 95% C.I.

Figure 3A shows the Grand Average ERP (electrodes P9 PO7—left; P10 PO8—right) for the four conditions: SymmetrySame, SymmetryNovel, AsymmetrySame, AsymmetryNovel. We computed two SPN difference waves based on matching conditions *SymmetrySame–AsymmetrySame* and *SymmetryNovel–AsymmetryNovel*. These are plotted in Fig. 3C,D respectively, along with 95% confidence intervals and individual-subject responses (dashed lines). Individual amplitude distributions, separately for each hemisphere and timewindow, are plotted in Fig. 3E,F. Figure 3B shows the topographic distribution of the difference waves in the two timewindows analysed.

A repeated measure ANOVA [Match (*SymmetrySame–AsymmetrySame*; *SymmetryNovel–AsymmetryNovel*) × Timewindow (250–350, 800–1000)] was conducted to assess SPN differences across timewindows and hemispheres. There was an interaction between the two factors (F(1,27) = 6.36, p = 0.02, η_G_^2^ = 0.05). The symmetry response emerged after approx. 200 ms from t0 and was sustained until after 500 ms (t2) for both difference waves (see Fig. 3C,D). The SPN in the 250–350 ms timewindow was significant for *Same* (t(27) = − 4.56, p < 0.001, dz = − 0.86; negative difference amplitude in 24/28 participants) and *Novel* (t(27) = − 5.31, p < 0.001, dz = − 1.0; 23/28 participants). In the timewindow 800–1000 ms the SPN emerged again for *Same* (t(27) = − 4.28, p < 0.001, dz = − 0.81; 20/28 participants) but not for *Novel* (t(27) = − 0.2, p = 0.85, dz = − 0.04; 13/28 participants).

### Discussion of experiment 1

The key result was the presence of a posterior negativity after t2. We assume this resulted from the amodal representation of symmetry when abstract shapes were partially occluded (t2). Importantly, this representation was history-dependent: only partial shapes that were recognised as previously seen as wholes elicited an SPN (i.e., *SymmetrySame*). At t2, the retinal image was the same for all conditions (i.e., an irregular polygon next to a rectangle). Although the rectangle should convey the experience of occlusion, symmetry could not be inferred based on the information available. This was demonstrated by similar ERPs in the *SymmetryNovel*, *AymmetrySame* and *AsymmetryNovel* conditions. The fact that *SymmetryNovel* did not elicit an SPN, showed that mere exposure to symmetry was not sufficient to affect the interpretation of new half-occluded shapes. Knowledge of the stimulus was critical for its features (i.e., symmetry) to be attributed to the amodally completed part.

There in an important caveat that requires consideration. We did not observe two discrete SPN waves in t0-t1 and t2. Instead, the difference wave was maintained for the whole epoch and upper-C.I. only briefly touched zero at the onset of the new stimulus in t2 (see Fig. 3). Therefore, the *SymmetrySame* SPN in t2 may not be an independent response to object-level symmetry. It might be attributed to low-level processes involving retinal image or visual persistence after the presentation of the fully-visible shape. The repetition of identical information along the central axis may have caused an *enhancement* (*priming*) *effect* on the ERP response in t2. Recent research has shown that when three symmetrical images are presented in succession, SPN amplitude increases^43,86,87^. This effect has been termed *SPN priming*^87^, and suggests that amplitude of the extra-striate symmetry response is partially determined by the immediately preceding stimulus. This response enhancement, however, is greater when the second exemplar is different from the first one^43^. In fact, Makin, et al.^87^ found no *SPN priming* for repeated presentation of identical exemplars. This category repetition advantage for non-identical symmetries is suggested to be related to getting new local information around a pre-encoded axis^43,87^ (see also behavioural findings^90^). In our experiment symmetry-primed novel shapes (i.e., *SymmetryNovel*) did not elicit any SPN response. On the contrary, we observed SPN only for identical second exemplars (i.e., *SymmetrySame*). It is thus unlikely that our result reflects *SPN priming*.

In Experiment 2 we addressed this potential confound. The shape in t2 was presented either above or below the fixation point. We expected a replication of Experiment 1, because amodal completion is a global phenomenon and should not rely on retinotopic match between the fully-visible and half-occluded stimulus. On the contrary, if SPN in t2 merely depended on repeated stimulation of same retinotopic visual areas, this should be erased in Experiment 2. For example, Makin et al.^87^ found no SPN priming when the repeating patterns changed retinal locations.

## Experiment 2

**Figure 4:**
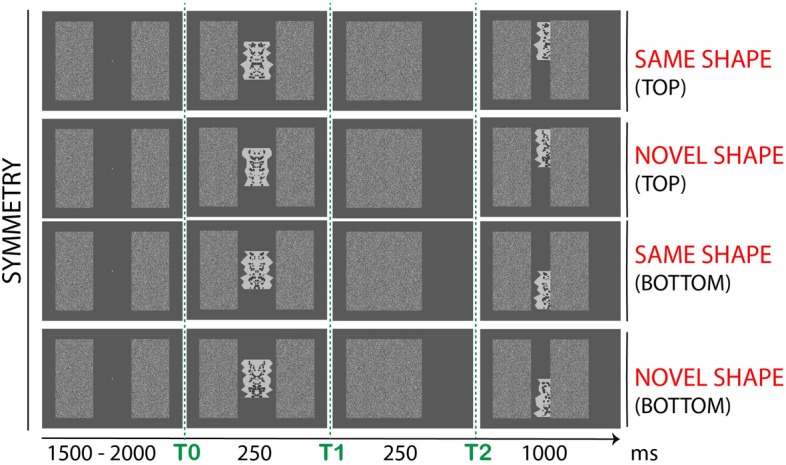
Example of stimuli and procedure in Experiment 2. The sequence of events was same as in Experiment 1, but position of the second polygon in t2 was either above or below the fixation dot (counterbalanced across trials). For simplicity, the figure illustrates only symmetric shapes and displacement of the right occluder. Same stimulus combinations were obtained for asymmetric shapes and left occluder (all counterbalanced across trials).

In Experiment 2, procedure was same as Experiment 1. The only difference was the position of the second polygon in t2. This could either be above or below the fixation dot (counterbalanced across trials; see Fig. 4). Participants were asked to keep eyes at fixation and matched the two shapes (in t0 and t2) as *Same* or *Different*. Here we tested whether the amodal representation of symmetry would resist changes in retinotopic correspondence between the fully-visible and half-occluded shape.

### Results

**Figure 5:**
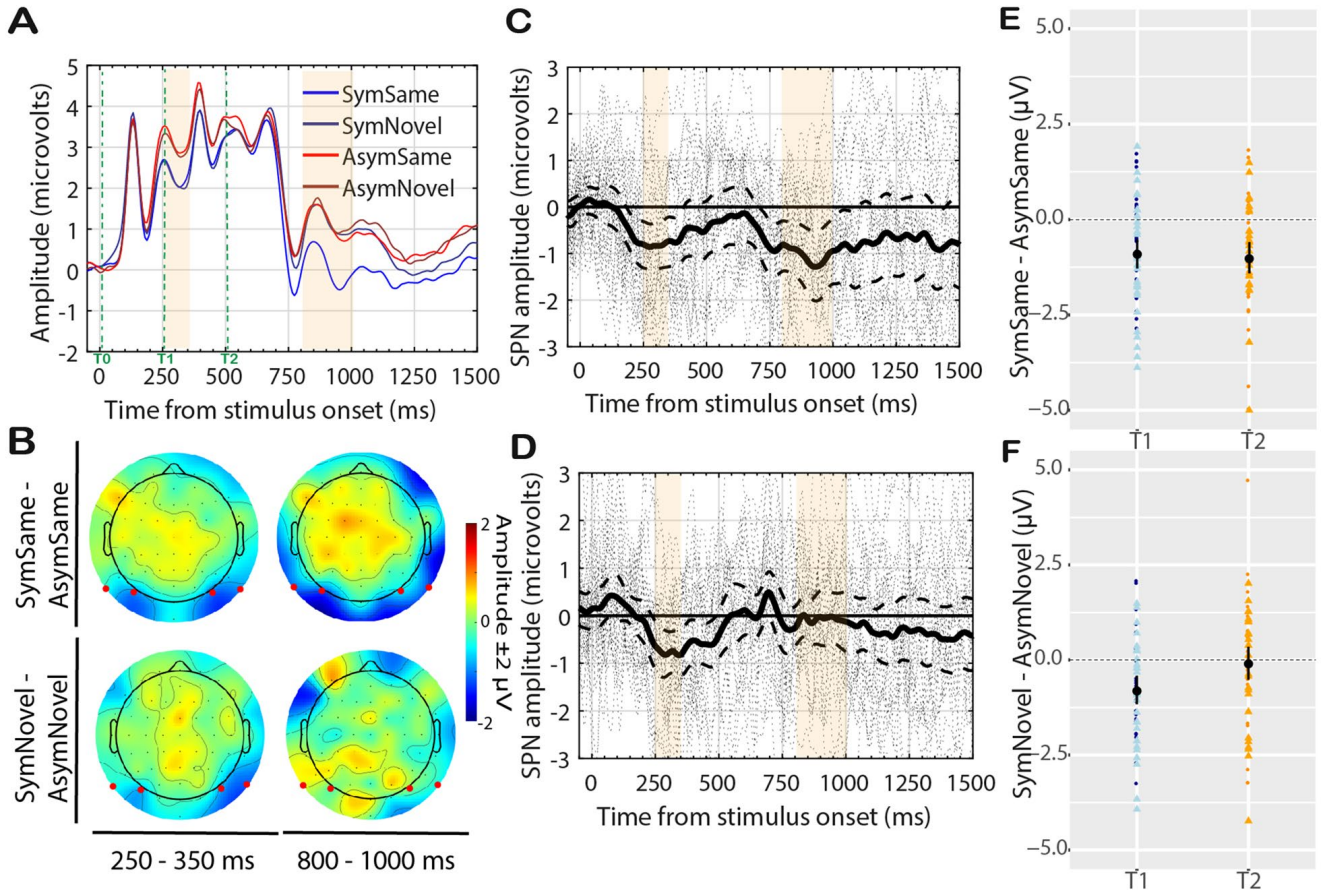
Experiment 2 Results. Conventions are the same as in Fig. 3.

Figure 5A–F shows the results of Experiment 2, which were similar to Experiment 1. The interaction Match × Timewindow was significant (F(1,27) = 7.6, p = 0.010, η_G_^2^ = 0.03) and explored below.

The response to symmetry emerged after approx. 250 ms and was sustained across part of the masking period (t1). The SPN in the 250–350 ms timewindow was significant for both *Same* (t(27) = − 3.71, p = 0.001, dz = − 0.70; 21/28 participants) and *Novel* (t(27) = − 3.65, p = 0.001, dz = 0.69; 22/28 participants) conditions. The SPN in the 800–1000 ms timewindow was significant for *Same* (t(27) = − 3.84, p = 0.001, dz = − 0.73; 23/28 participants) but not in the *Novel* condition (t(27) = − 0.32, p = 0.75, dz = − 0.06; 14/28 participants).

### Discussion Experiment 2

The results of Experiment 2 replicated those of Experiment 1. Here the SPN for the full shape was less sustained, and C.I. reached zero at approx. 500 ms for both difference waves (Fig. 5C,D). At t2, a new discrete SPN was elicited for the *Same* but not for the *Novel* condition. This experiment strengthened the conclusion that global amodal completion of symmetry can be achieved based on prior exposure and is the cause of the later SPN. Because of the positional change in t2, it is unlikely that responses at t2 reflect *SPN priming*.

## Experiment 3

**Figure 6:**
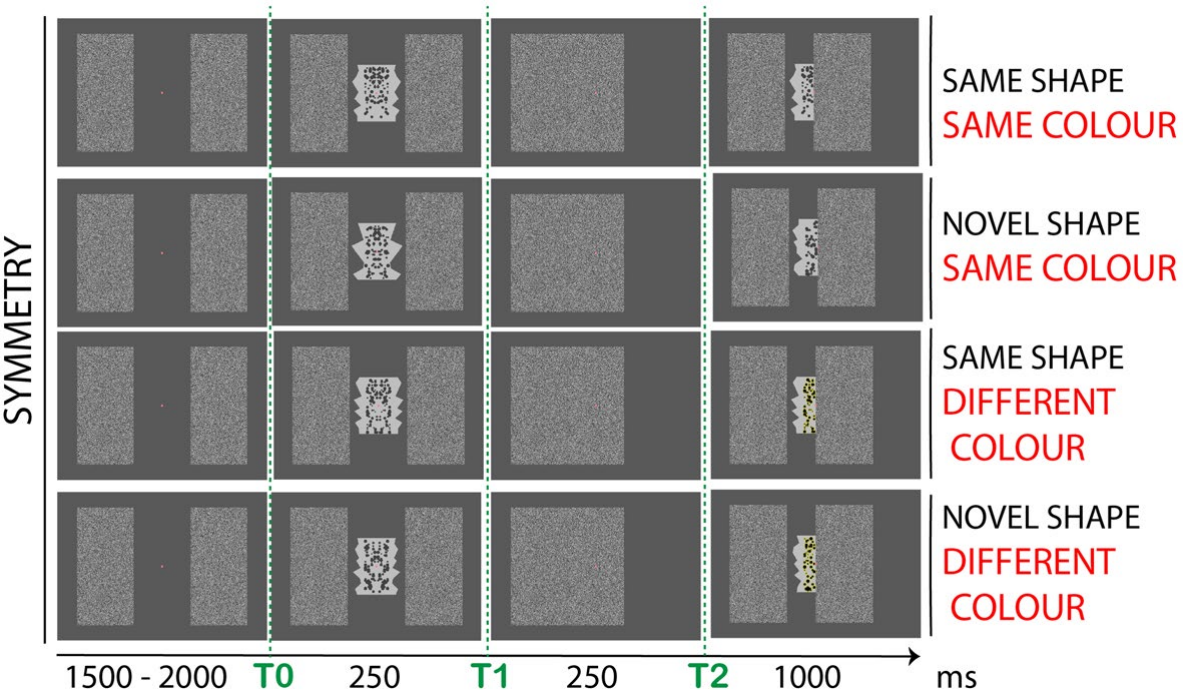
Experiment 3 experimental design was similar to Experiment 1. The shape in t2 could be either same as previously seen or novel. The additional factor was colour of the internal dot pattern, which could also be *Same* or *Different* in the two timewindows (counterbalanced across trials). Participants matched the colours and ignored the shapes. The figure illustrates the possible stimulus combination only for symmetry and right occluder displacement. The same combinations were presented for asymmetry and left occluder (counterbalanced across trials). The difference in colour was subtle (dark grey or black) to ensure participants’ engagement with the task. For illustrative purposes yellow contour lines have been added to the dot patterns (bottom rows) to highlight the difference in colour, but these were not present in the actual experiment.

Experiment 3 investigated whether the amodal representation of symmetry requires participants to actively match the two shapes, or whether it emerges automatically even when other stimulus dimensions are attended. The design was similar to previous experiments, but participants attended to the colour of the internal dot pattern (dark grey or black). The colour change was subtle to ensure participants’ engagement with the stimuli. They reported whether colour in t2 was *Same* as or *Different* to t0 (see Fig. 6).

Recent research shows that responses to object-level symmetry are formed only when participants attend to regularity; when participants attend to other dimensions (e.g., colour) the brain responds only to symmetry in the image^30,46,51^. We observed object-level SPN in Experiment 1 and 2, despite participants not attending to symmetry. In these two experiments, however, a representation of the shape must be retained in Visual Sensory Memory (vSM) in order to do the task. This was not necessary in Experiment 3. We expected no global completion of symmetry in the *SymmetrySame* condition in t2 in absence of active shape recognition (but we expected a symmetry-SPN in the first timewindow).

### Results

**Figure 7:**
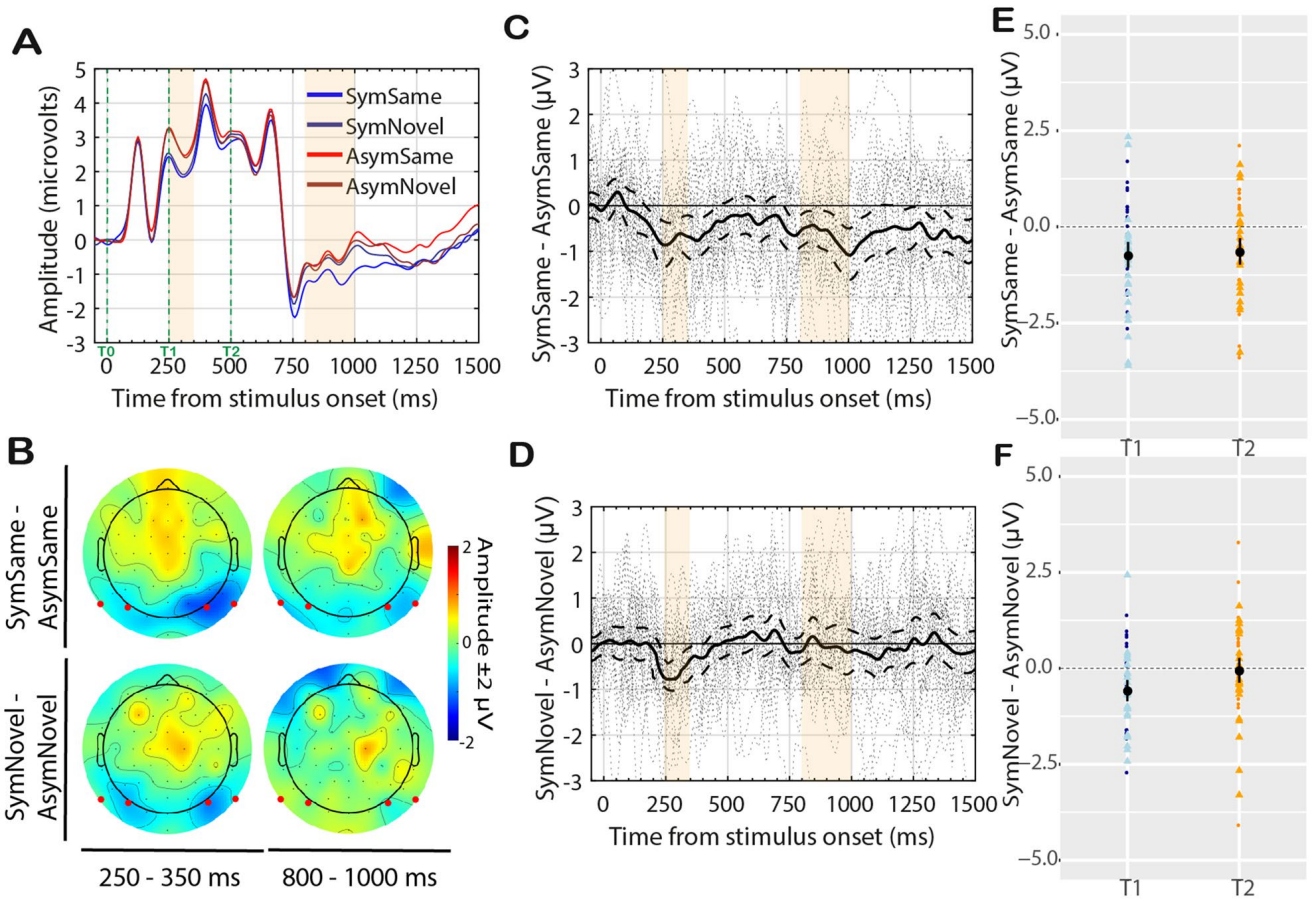
Experiment 3 Results. Conventions are the same as in Figs. 3 and 5.

Figure 7A–F shows the results for Experiment 3. Note that data were analysed in the same way as for Experiment 1 and 2; *Colour* was not included as a factor in the analysis.

The interaction between Match × Timewindow was not significant (F(27) = 1.29, p = 0.27, η_G_^2^ = 0.01). However, there was an SPN in the *Same* condition after t2 (t(27) = − 3.32, p = 0.003, dz = − 0.63; 23/28 participants; see Fig. 7C). There was no SPN in the *Novel* condition after t2 (t(27) = − 0.4, p = 0.70, dz = − 0.07; 15/28 participants; see Fig. 7D). Results for the 250–350 ms timewindow were similar to Experiment 1 and 2 as expected. The SPN was significant for both *Same* (t(27) = − 5.00, p < 0.001 , dz = − 0.94; 23/28 participants) and *Novel* (t(27) = − 3.58, p = 0.001, dz = 0.68; 21/28 participants).

### Discussion Experiment 3

We observed a reduced but significant SPN for *SymmetrySame* in t2. Recent findings have confirmed no object-level symmetry SPN when other stimulus dimensions (i.e., colour) were attended^30,46,51^. It has been argued that extracting symmetry at object-level is a demanding perceptual operation and this operation does not take place when symmetry is not relevant to the primary task. It is thus interesting that the amodal symmetry representation was present in the current study.

Image-symmetry in t0 triggered an SPN (as expected), meaning that its representation was formed in the brain. Possibly this representation was retained in vSM for a short interval, despite attention being directed to colour. When part of this learned information was presented again (in t2), it automatically reactivated the perception of the occluded part. Note that we used subtle colour difference between the two shapes so to ensure participants would engage attention to the stimuli (which could be avoided with greater colour difference, e.g. grey vs red).

Another possibility (already discussed for Experiment 1) is that residual symmetry-activity carried over after offset of image-symmetry and reinforced a response when identical information was instantiated again. Experiment 4 was designed to test the presence of this residual carryover activity.

## Experiment 4

**Figure 8:**
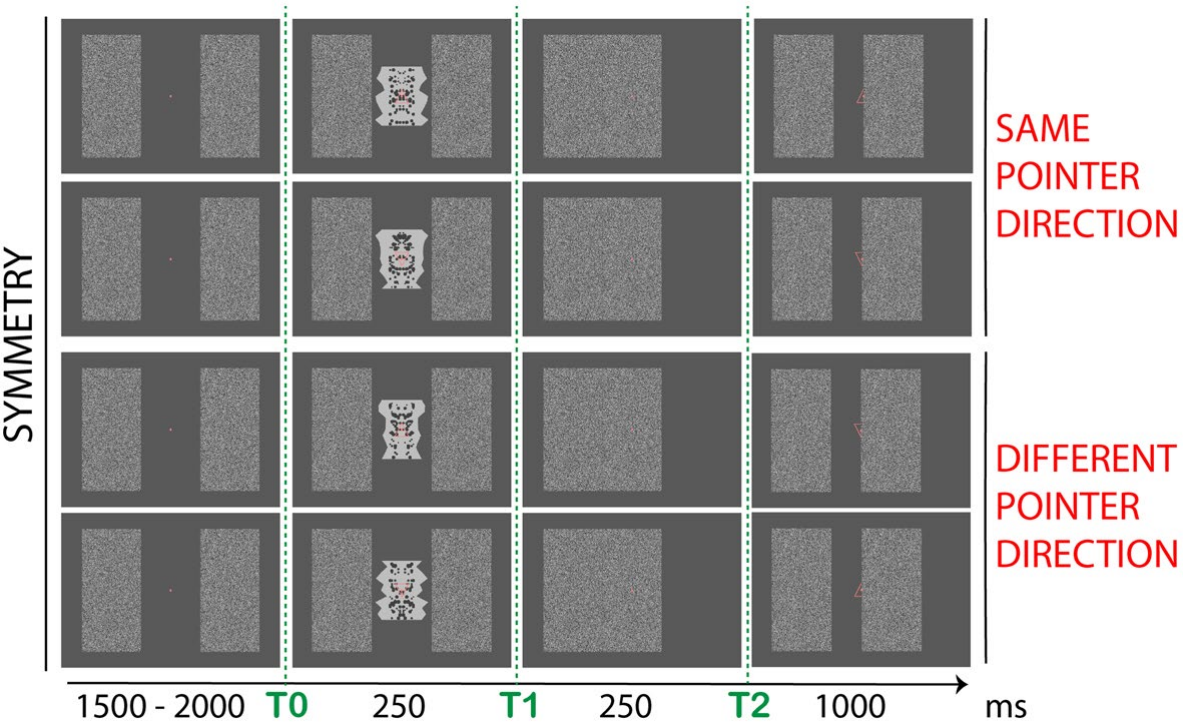
Experiment 4. The design was similar to the other experiments. A red triangle was presented at fixation, superimposed to the symmetric shape in t0. It pointed either upwards or downwards. In t2 no shape was presented behind the occluder; only the triangle pointing towards either the *Same* or *Different* direction (participants attended the triangle/pointer direction). In the figure only combinations for symmetry and right occluder displacement are shown. Same combinations were obtained for asymmetry and left occluder (all counterbalanced across trials).

The design of Experiment 4 was similar to previous experiments, but only full shapes were presented at t0 and no occluded shape was shown at t2 (see Fig. 8). Participants attended the orientation of a triangle (i.e. upward or downward), superimposed to the shape at fixation. The triangle pointed towards either *Same* or *Different* direction in the two time-windows. Image-symmetry elicits an SPN when attention is directed to other overlapping stimuli, either in same or different modality^44^. We thus expected an SPN at t0. The absence of shape at t2 allowed to test for baseline residual activity in that timewindow. In this experiment a simple Symmetry–Asymmetry difference was measured.

### Results

The data were analysed by looking at the difference Symmetry–Asymmetry, because there was no shape presented in t2. The pointer direction was not included in the analysis.

**Figure 9:**
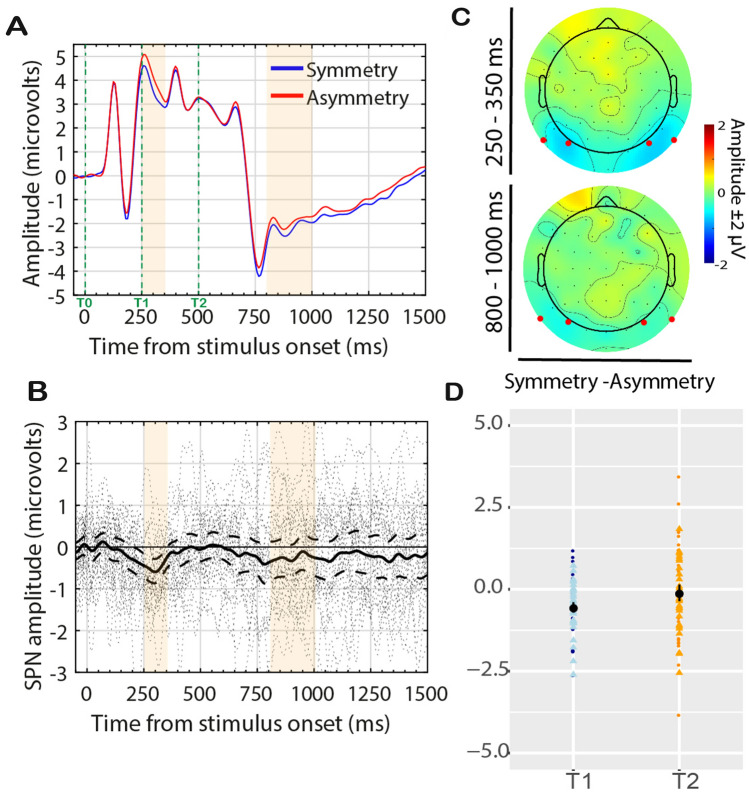
Experiment 4 results. Conventions are the same as Figs. 3, 5 and 7, except there is no Same and Novel distinction in Experiment 4.

Figure 9A shows the Grand Average ERPs and Fig. 9B *Symmetry*–*Asymmetry* difference wave with 95% C.I. and individual waves. An SPN was recorded in the first time-window, emerging after approx. 250 ms (t(27) = − 4.47, p < 0.001 , dz = − 0.85; 22/28 participants). No residual symmetry-related activity was observed in t2 (t(27) = − 1.12, p = 0.27, dz = − 0.21; 16/28 participants; see Fig. 3B). No difference between hemispheres was observed at any timewindow (see Fig. 3B).

### Discussion Experiment 4

We observed automatic response to image-symmetry at t0 with short presentations (250 ms). This confirms the presence of a default task-independent high-sensitivity to symmetry in the extrastriate cortex^44^. Importantly, there was no evidence of residual symmetry-specific activity carried over across the epoch. The posterior negativity observed at t2 in previous experiments thus reflected a newly generated completion response, based on recognition of the partial information of the half-occluded shapes.

## General discussion

In the case of half-occluded familiar objects (e.g. a face or a car seen in front view), object-knowledge may favour global completion based on symmetry. For unfamiliar shapes, the completion of the invisible half requires contextual information, e.g. prior exposure to the object in full-view. This study showed ERP evidence of history-based amodal completion of symmetry (object-level representation) in half-occluded abstract shapes.

We designed a paradigm that gave the impression of a large rectangle moving from a flanking position to cover a shape for a short interval. The occluder then shifted partly to the side to reveal only half of the hidden shape. This half was either the *Same* as that of the previously seen shape or *Novel* (participants reported whether the two stimuli from the two intervals matched). Symmetry was task-irrelevant in all experiments because stimuli could match whether they were symmetrical (in one condition) or not (in another).

The first finding is that in all four experiments we observed automatic SPN to symmetry in the image (t0-t1) with very brief presentations (i.e., 250 ms) followed by a mask (the occluder). Task independent responses to symmetry have been previously reported, when participants attended to different properties (e.g., colour^43,91^, number of closed regions^92^, infrequent features^48^) either in the same or different sensory modality^44,47^. Bertamini et al.^43^ used short presentation (i.e., 500 ms) and observed an automatic SPN. Here we further demonstrate the brain’s high sensitivity to symmetry. This result also shows that the sustained nature of the component is quite flexible and may reflect internal dynamics of the symmetry-sensitive network (e.g. attentional processes, retention of symmetry representation in working memory, see^43^). As other studies have suggested, the first part of the component is likely to reflect actual perceptual representation processes^39,51,89^.

The most important finding was a selective symmetry-negativity after t2. This may reflect a history-dependent amodal completion of a symmetrical object behind the occluder. Importantly, the amodal representation of symmetry was achieved only when half-occluded shapes were recognised as *Same* as those previously seen (i.e., *SymmetrySame*–*AsymmetrySame* difference). When occlusion covers a large proportion of the shape (up to its vertical midline), local completion is preferred and the global symmetric representation of the object is not achieved^62,71^. This was evident in the fact that novel shapes were not automatically completed based on symmetry (i.e., *SymmetryNovel*–*AsymmetryNovel*). Our study showed that recognition of the previously-seen object drove the global interpretation of the half-occluded shape. This is in line with literature showing strong prior exposure effects on amodal completion^72,74,76,79,82^, especially in cases with ambiguous competing multiple interpretations^79^.

We considered the possibility that the posterior negativity at t2 may reflect a response enhancement caused by the repeated stimulation of the same retinotopic regions in V1 along the central axis of symmetry. This could explain the unexpected result in Experiment 3, where negativity at t2 for SymmetrySame was observed despite the fact that shape similarity was not attended. Perhaps residual symmetry-related activity was maintained and reinforced when the same local information was stimulated again. Previous research showed that presenting symmetric patterns sequentially caused SPN enhancement^43,87^. However, in Experiment 2, shapes at t2 were presented in a different location (either above or below the fixation point). If SPN in t2 depended on information repetition along a pre-encoded axis, it should not appear when this information changed retinal location. Contrary to this interpretation, results were similar to Experiment 1. In addition, Experiment 4 showed no symmetry response being carried over throughout the t2 timeframe.

Another possible interpretation is that the negativity at t2 reflects change-detection in visual short-term memory. It has been found that a sustained posterior positivity, peaking at 400 ms from stimulus onset, is generated when participants detect changes between two sequential configurations^93,94^. This is unlikely here, because participants correctly detected similarity between asymmetric and occluded shapes but there was no difference between these conditions (i.e., *AsymmetrySame* and *AsymmetryNovel*).

Finally, this study cannot tell whether exposure influenced the completion process itself or acted to reinterpret the figure after it was perceived. We thus cannot say whether SPN in t2 indexed a genuine perceptual completion process or some later decision stage^82^. If the latter was the case, we may have expected exposure to symmetry to influence the interpretation of any occluded shape regardless of its similarity. However, we observed no SPN for *SymmetryNovel* condition (but see Supplementary_Material_1, reporting ERPs from trials where incorrect responses were made in Experiment 1 and 2. The SPN for *SymmetrySame* was not elicited in t2. On the contrary there was a tendency response to symmetry for *SymmetryNovel*).

It would be interesting to extend this investigation to other forms of regularity (e.g. translation symmetry; 180-degrees rotation symmetry). The SPN amplitude for translation and 180-degrees rotation symmetry—in the image—is reduced compared to one-axis bilateral reflection symmetry, as a function of their perceptual salience^24,39,48^. Would prior exposure to different levels of regularity lead to different SPN amplitudes at t2? This would support the hypothesis of a genuine perceptual completion process^82^. Alternatively, a similar response might be generated for all conditions, reflecting post-perceptual processes related to the task.

In this study we used onefold reflection symmetry, occluded up to the central vertical axis of symmetry. Shape regularity per se could not act as a global cue for amodal completion. If some of the symmetry of the partly-occluded shape was visible, global completion might proceed automatically without the need of prior exposure. One approach could be to use twofold reflection symmetry with different levels of occlusion (i.e. ¼, ½, ¾). We know that the SPN’s amplitude scales parametrically with the amount of regularity in the image^26,39^. Would the extra-striate symmetry-network merely respond to image symmetry, or would it be invariant to occlusion (i.e. object’s symmetry being represented to same extent in full-view as in ½ and ¼ occlusion)?

In conclusion, in this study we found evidence of a representation of symmetry generated by the amodal completion of partially occluded symmetrical shapes. This adds to the recent literature showing that the representation of symmetry can exist in the brain at a non-retinotopic global level^30,46,51^ (see also behavioural evidence^90,95–97^). The amodal symmetry was indexed by a SPN-like response over extrastriate areas, in line with literature showing amodal completion is achieved at the level of LOC^56,57^.

Our results support behavioural^90,95–97^ and recent neuroscientific evidence^30,46,51,52^ of global processes for symmetry representation. We propose that classic models^6,11,12,20–25^, which emphasise early responses to pairwise correlations in the image, should be updated to consider the flexibility of mechanisms for symmetry perception. We conclude that object-knowledge influences the extrastriate representation of the object’s (reflection) symmetry in static visual occlusion.

## Method

### Participants

One hundred and twelve (N = 112) participants took part in this study (median age = 19.5, range = 53–18; 26 males; 12 left-handed), divided into groups of twenty-eight (N = 28) participants for each experiment. Sample size was selected a priori for consistency with previous studies assessing non-retinotopic representation of symmetry (i.e., Rampone et al., 2019). Participants had normal or corrected-to-normal vision. Some received course credit upon completion of the study. The study was approved by the University of Liverpool Ethics Committee (reference: 2122). Participants signed an informed consent form in order to take part in the study. The study was conducted in accordance with the Declaration of Helsinki (although it was not pre-registered, which is required by point 35 of the 2008 revision).

### Apparatus

EEG activity was recorded using a BioSemi Active-Two amplifier in an electrically shielded and darkened room. EEG data were sampled continuously at 512 Hz from 64 scalp electrodes embedded in an elasticised cap arranged according to the standard international 10–20 system. To detect blinks and eye movements, vertical bipolar electrodes (VEOG) were positioned above and below the right eye. Horizontal bipolar electrodes (HEOG) were positioned on the outer canthi of both eyes. Stimuli and experiment were programmed using PsychoPy software (coder view)^98^ and presented on a 29 × 51 cm LCD monitor (60 Hz refresh rate). Participants were positioned 57 cm from the monitor with their head stabilised in a chin rest.

### Stimuli

Stimuli were similar to those used in Rampone et al.^51^. They consisted of complex polygons (light-grey: RGB [0.5, 0.5, 0.5], luminance 78.5 cd/m^2^) containing a dot pattern (dark-grey: RGB [− 0.5, − 0.5, − 0.5], luminance 14.5 cd/m^2^). The stimuli were presented on a mid-grey background (RGB [− 0.3, − 0.3, − 0.3], luminance 39.0 cd/m^2^) (RGB colour space is expressed as deviations from grey ranging between − 1 and 1^98^). All shapes were generated afresh on each trial. No participant ever saw the same pattern twice. Polygons were generated by creating one half first, with a random-walk algorithm (12 inward and outward turns). The second half was either generated independently (asymmetry condition) or it was mirrored (symmetry condition). Each turn was spaced approx. 0.7° longitudinally and had a maximum and minimum transverse displacement of approx. ± 0.8°. Top and bottom vertices were connected with straight lines to form a closed polygon (size approx. 7.5° × 4.8°). The dot pattern drawn inside each polygon (half of stimulus shape) was formed by approximately 40 dots (mean number of dots = 41.2, SD = 4.2). Dots’ radius varied randomly between 0.08° and 0.24°. Dots were placed in random positions within a matrix of 119 cells and confined within an area of 1.6° × 3°. The dot patter was also either symmetric or asymmetric as its polygon shape.

The occluder stimuli were two rectangles bearing a black-and-white grating-texture (luminance 46 cd/m^2^) with size approx. 17.4° × 9.6°. The rectangles were placed both at the left and right on the screen at ± 9.6° from central fixation. On each trial, one of the two occluders moved towards fixation (i.e., aligned with central midline) at t1; then moved ± 4.8° towards original position so that its edge was aligned at the central midline with the edge of half-polygon in t2 (see Fig. 2).

#### Stimuli variation in the different experiments

Stimuli were the same in Experiment 2; the only difference was that the half-polygon in t2 were presented either upward or downward respect to central fixation (i.e., either top or bottom of the polygon aligned with central midline; see Fig. 4). In Experiment 3 the colour factor was determined by the colour of the internal dot pattern. This could be either same as in other experiments (i.e., dark-grey: RGB [− 0.5, − 0.5, − 0.5]) or black (RGB [− 1, − 1, − 1]; see Fig. 6). In Experiment 4 a triangle frame of size 2.1° × 2.1° and colour pink-red (RGB [1, 0, 0]) was superimposed at the centre of the polygon shape at t1 and half-covered (and presented in isolation) in t2 (see Fig. 8).

### Procedure and design

In all experiments, participants completed a practice before starting the experiment to familiarise with the task. One practice block included 32 trials and a response-feedback (i.e. sound for incorrect responses), which was not present in the experimental block. Participants had the possibility to repeat the practice as many times as they wished until they reached confidence. This means that each participant might receive different amount of training, depending on task difficulty and individual performance. They were required to maintain fixation and refrain from blinking for the whole trial duration. Breaks were provided during the experiment to allow participants to rest.

The sequence of events of Experiment 1 is described in Fig. 2. A baseline interval of 1500 ms with only the fixation dot and the two occluders on screen was followed by the appearance of the stimulus shape (t0), either symmetry or asymmetry. This stayed on the screen for 250 ms, then one of the two occluders (either the left or right one, counterbalanced across trials) moved at fixation covering the shape entirely (t1) for 250 ms. The same occluder moved again towards original position but stopped at the central fixation midline, which revealed only half of the shape underneath (for 1000 ms; t_2_). This could be either the same shape as presented before or a novel shape. Finally, the occluder returned at the original position and a response message appeared in the central space between the two occluder bars. Participants were prompted to report whether the shape presented at t2 was *Same* or *Novel* (the position order “*Same Novel*” or “*Novel Same*” was counterbalanced across trials to avoid artifacts produced by preparation of a motor response during the stimulus presentation period). This means that participants never had to pay attention to stimulus symmetry (in fact symmetry was never mentioned until final debrief). Participants entered a response, by pressing either ‘A’ or ‘L’ button of the computer keyboard with their left or right index fingers. They were explicitly informed that responses needed to be as accurate as possible, whilst response speed was not measured. This also minimized motor responses artifacts.

The full design of the experiment was 2 (*shape regularity*: symmetry, asymmetry) × 2 (*shape match*: same, novel) × 2 (*occluder direction*: left, right). Only *shape regularity* and *shape match* were considered for the analysis, giving four possible combinations: SymmetrySame (symmetry in t1 and the same stimulus in t2), SymmetryNovel (symmetry in t1 and a novel stimulus in t2), AsymmetrySame (asymmetry in t1 and the same stimulus in t2), AsymmetryNovel (asymmetry in t1 and a novel stimulus in t2); see Fig. 2. The experiment consisted of a total of 320 trials (80 × 4 sub-conditions).

Please note that the terminology used in the description reflects the percept of (dynamic) occlusion. There is no actual physical occlusion in these stimuli. Hence, events within a trial may be described as follows. In the first interval, a polygon shape (either symmetric or asymmetric) was shown at center of fixation flanked by two rectangles. In the second interval, one of the two rectangles was shown at fixation adjacent to the other rectangle. In the third interval, an irregular polygon was shown at fixation next to a rectangle. This could match half of the polygon shape presented in the first interval or be unrelated. Similarly, when we refer to movement of the occluder, the motion is implied by a change of location. We exclude an effect of apparent motion on the results. The same motion effect would be present in all conditions and cancelled out by averaging and computing a relative measure of the brain response (i.e. symmetry–asymmetry). Please find video illustrating the sequence of events in a trial in Supplementary_Material_2.

#### Procedure variations in the different experiments

In Experiment 2, procedure and design were same as Experiment 1. The only difference was that the second polygons at t2 could either be presented above or below the fixation dot (counterbalanced across trials; see Fig. 4). Experiment 3 used a similar procedure, although participants matched the two stimuli by colour of the internal dot pattern. Participants had to report whether the colour of the inner dot pattern in the second polygon was *Same* or *Different* as in the first polygon shape. The experiment design was 2 (*shape regularity*: symmetry, asymmetry) × 2 (*shape match*: same, novel) × 2 (*colour match*: same, different) × 2 (*colour order*: darker first, lighter first) × 2 (*occluder direction*: left, right). The number of trials was the same and the same four conditions were considered for the analysis (i.e., *shape regularity* and *shape match).* In Experiment 4 participants matched the pointing direction (upwards, downwards) of a triangle frame presented in the centre of the screen. In t1 this was superimposed to the polygon shape, in t2 this was shown in isolation and half covered by the occluder. No half-polygon was presented in t2. Participants reported whether the triangles both pointed to *Same* direction (e.g., both pointing upwards) or to *Different* direction. The design was 2 (*shape regularity*: symmetry, asymmetry) × 2 (*pointer match*: same, different) × 2 (*pointer order*: upward first, downward first) × 2 (*occluder direction*: left, right). The number of trials was the same as the other experiments, however only *shape regularity* was considered for analysis. Note that in Experiment 3 and 4 the label *Novel* was replaced with *Different*, which was deemed more appropriate to indicate the change of a stimulus feature. This was decided after running a pilot version of the experiments and debriefing participants.

### Data analysis

EEG data was processed using the EEGLAB v2019.1 toolbox in MATLAB^99^ and the same criteria as in Rampone et al.,^51,52^. Data was first imported using reference Cz then referenced to a scalp average (using *pop_reref*). This was then followed by filtering (using *pop_eegfiltnew;* high-pass 0.1 Hz and low-pass 25 Hz) and down-sampling to 128 Hz (using *pop_resample*). We segmented the data into − 1 to 2 s epochs (*pop_epochs*) and set to baseline (− 200 ms; *pop_rmbase*). Independent Components Analysis (ICA) was used (*pop_runica*) to remove oculomotor and other gross artefacts. After ICA, trials where amplitude exceed +/− 100 μV at any electrode were excluded (*pop_eegthresh*). Moreover, trials where participants entered incorrect response were excluded from the analysis. Therefore, we included only trials where the correct match between second and first polygon shapes was made.

#### ICA and trials rejections for each experiment

In Experiment 1 on average 12 (SD = 4.9) out of 64 components were rejected (min = 1, max = 20). After data cleaning, the average number of trials included was 64.6 (SD = 8.7) for SymmetrySame, 66.9 (SD = 6.9) for SymmetryNovel, 58.4 (SD = 11.6) for AsymmetrySame, 59.7 (SD = 9.2) for AsymmetryNovel. In Experiment 2 on average 11 components (SD = 3.5; min = 5 max = 17) were removed. Average number of trials included was 58.1 (SD = 18.6) for SymmetrySame, 59.4 (SD = 18.8) SymmetryNovel, 54.8 (SD = 19.1) AsymmetrySame, 50.0 (SD = 18.5) AsymmetryNovel. In Experiment 3 on average 12 (SD = 4.5, min = 3 max = 19) components were removed and number of trials included was 74.4 (SD = 6.3) SymmetrySame, 74 (SD = 6.4) SymmetryNovel, 74.6 (SD = 5.3) AsymmetrySame, 73.8 (SD = 6.7) AsymmetryNovel. In Experiment 4 components removed were 10.6 (SD = 4.3, min = 4 max = 19) and trials included were 72.32 (SD = 8.3) SymmetrySame, 72.75 (SD = 8.8) SymmetryNovel, 73.14 (SD = 7.4) AsymmetrySame, 73.7 (SD = 7.9) AsymmetryNovel.

The processed ERP data were analysed in R. For the analysis, we used a cluster of posterior electrodes (left hemisphere: P9, PO7; right hemisphere: P10, PO8; same as Rampone et al.^51^). PO7/PO8 best represent the topographical distribution of the SPN^26,37–39,42^. We were interested in measuring the response to symmetry after the presentation of the full polygon shape in t0 and after the onset of the second polygon in t2. The timewindows analysed were decided a priori. The first time-window 250 ms–350 ms was selected based on literature showing that the SPN starts at approx. 250–300 ms from stimulus onset^42^; the end point was admittedly selected post hoc based on the presence of a P1-like positive peak starting at 350 ms, possibly generated by the onset of the occluder at fixation (note the peak was observed in all four experiments with same latency, Figs. 3A, 5A, 7A, 9A). The second time-window 800–1000 ms (i.e., 300–500 ms from t_2_) was selected a priori based on Rampone et al.^51^. The response to symmetry (i.e., the SPN) is a relative measure and is best represented as a difference from 0% symmetry (i.e., the asymmetry condition). Therefore, we computed the differences *SymmetrySame–AsymmetrySame* and *SymmetryNovel–AsymmetryNovel* and used these in the analysis as a 2-level factor. The difference ERPs data were normally distributed (Shapiro–Wilk tests p_s_ > 0.05; with exceptions: *SymmetrySame—AsymmetrySame* T2 in Experiment 2, W(27) = 0.97, p < 0.001; *SymmetryNovel–AsymmetryNovel* T2 in Experiment 3, W(27) = 0.93, p = 0.004) ERP between symmetry–asymmetry was calculated and used for the analysis. We had different predictions for the two selected timewindows, therefore these were analysed separately with repeated measure ANOVA (with R package ezANOVA) and t-test. Generalised eta squared (η_G_^2^)^100^ and Cohen’s d were used to report effect sizes.

Topographic maps of the difference ERPs (shown in Figs. 1B,F, 3B, 5B, 7B, 9C) were generated using the function *topoplot()* of the EEGLAB toolbox.

#### Proportion of correct (behavioural) responses for each experiment

In Experiment 1 median proportion of correct responses was 85 (range = 95–66) SymmetrySame, 77.5 (range = 94–56) SymmetryNovel, 95 (range = 100–73) AsymmetrySame, 90.6 (range = 100–69). In Experiment 2 this was: SymmetrySame, median 76 (range 96–55); SymmetryNovel, median 72 (range 94–54); AsymmetrySame, median 87 (range 98–70); AsymmetryNovel, median 88 (range 98–70). In Experiment 3 this was: Same Colour, median 99 (range 100–48); Different Colour, median 98 (range 100–52). In Experiment 4 this was: Same Pointer, median 98 (range 100–88); Different Pointer, median 97 (100–86).

Materials for re-running the experiments and re-analysing the results are available on Open Science Framework in “The complete Liverpool SPN catalogue”, along with other data from experiments about perception of symmetry (Project 31, https://osf.io/2sncj/). The study was not preregistered, but all hypotheses and analyses were planned a priori.

## Supplementary Information

Supplementary Information 1.

Supplementary Information 2.
