# Supplementary material for: Electrophysiological evidence of the amodal representation of symmetry in extrastriate areas
Source: Sci Rep. 2022 Jan 21;12:1180. doi: 10.1038/s41598-021-04501-3 (PMC8783022; doi:10.1038/s41598-021-04501-3)
Supplement: Supplementary file 1 — Supplementary Information 1. [file 41598_2021_4501_MOESM1_ESM.docx]

**Supplementary Material 1**

**Analysis of ERP responses in Incorrect Trials – Experiment 1 and 2**

We analysed ERP responses from trials where an incorrect response was entered. Note that only trials with correct responses were included in the main analysis in the paper. We expected to see no response to *SymmetrySame* in t2 in the incorrect trials. This because amodal completion of symmetry was expected to emerge from the correct recognition of the occluded shape in t2.

Note that there was not a large number of incorrect trials and data from some participants are missing for some conditions (due to 100% correct responses). The average number of trials per participant was: SymmetrySame 6.8 (SD ± 6.6), SymmetryNovel 8.2 (± 6.4), AsymmetrySame 13.6 (±9.8) and AsymmetryNovel 14.1 (±10.3). Therefore we limit ourselves to report descriptive plots of results from Experiment 1 (Figure 1 A,B) and Experiment 2 (Figure 1 C,D).


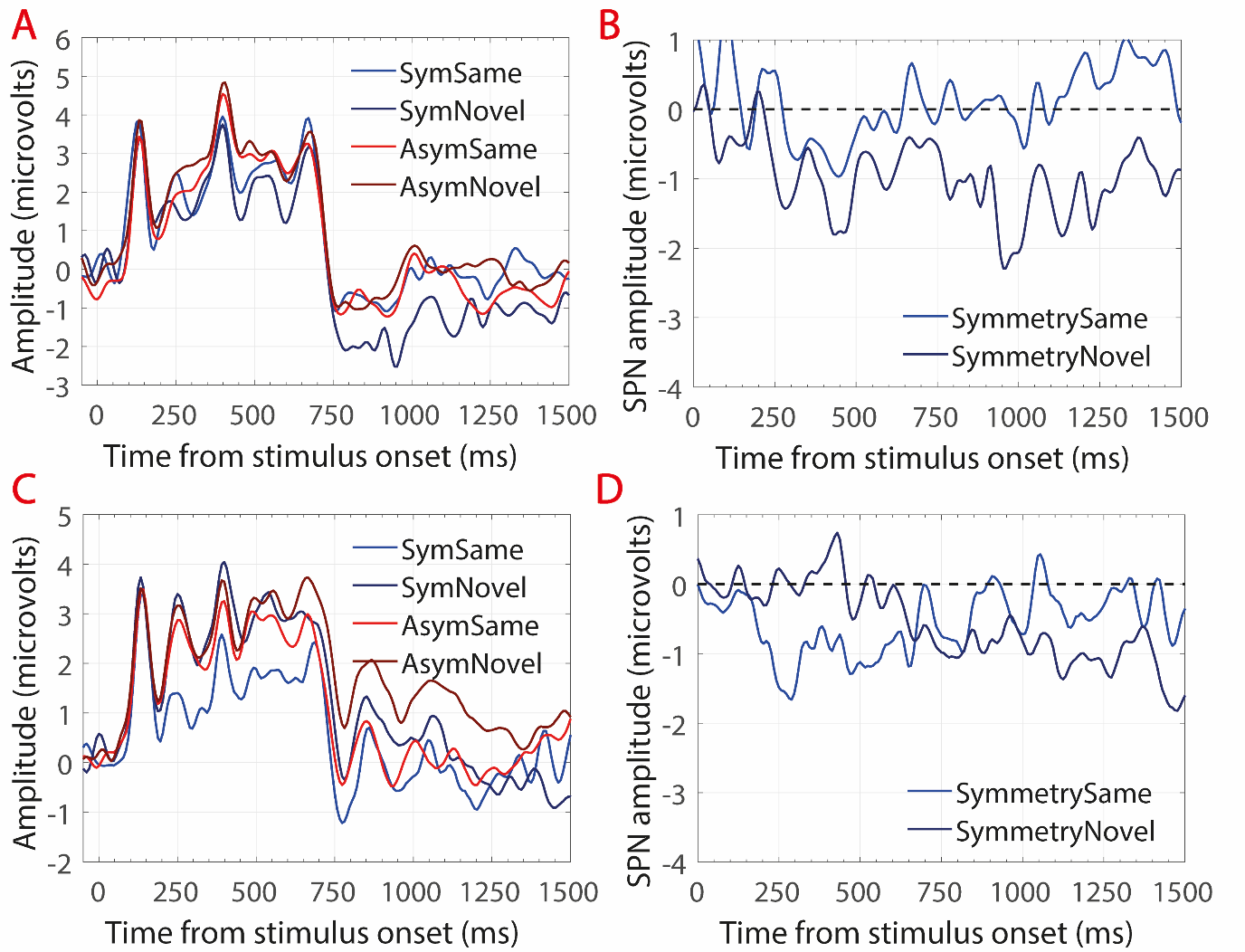


**Figure 1.** (A) Grand Average ERPs for the four conditions in Experiment 1. (B) Difference waves for *SymmetrySame* – *Asymmetry Same* and *SymmetryNovel* – *AsymmetryNovel* in Experiment 1. (C) Grand Average ERPs and (D) Difference waves in Experiment 2.

Fully-visible shapes elicited a symmetry response in t0-t1, which is expected for incorrect trials since this should be automatic (no attention to symmetry was required). Oddly, there is no response for SymmetryNovel condition in Experiment 2 in this timeframe.

There is no evidence for *SymmetrySame* eliciting an SPN at t2 when shapes were not correctly recognised. On the contrary, there is a tendency of stronger negativity for *SymmetryNovel* (which was erroneously classified as *Same*) in Experiment 1 (Figure 1A and 1B). However, this was not replicated in Experiment 2. This tendency might suggest that symmetry representation might be formed when participants believe they have seen the (symmetric) shape before.

This brings in a potential confound, which we have discussed in the Discussion section. There might be the possibility that *exposure* did not directly influence the perceptual completion process itself. On the contrary, it may have acted on some later decisional process which influenced the interpretation of the figure after it was perceived.

If this was the case, however, one would expect *exposure* to symmetry to influence the interpretation of novel occluded shapes too (i.e. SymmetryNovel). However, we did not observe this in the results reported in the manuscript. Possibly, if participants were able to tell the shape was new (correct responses), they treated it as an independent entity separate from the context. Instead if they mistakenly believed they had previously seen the shape (incorrect response), they attributed the symmetry information to a new exemplar. This elicited a global completion of the figure based on symmetry.

The above is only a speculation. Our study was not designed to address this issue, which should be considered in future research.
