# Supplementary material for: Electrophysiological evidence of the amodal representation of symmetry in extrastriate areas
Source: Sci Rep. 2022 Jan 21;12:1180. doi: 10.1038/s41598-021-04501-3 (PMC8783022; doi:10.1038/s41598-021-04501-3)
Supplement: Supplementary file 2 — Supplementary Information 2. [file 41598_2021_4501_MOESM2_ESM.zip › read me.docx]

Video Recording of the sequence of events in a trial in Experiment 1.

**Symmetry Same_right occluder:** Symmetry shown in t1, and same shape shown in t2. Displacement of right occluder
